# National pharmacological treatment trends for ankylosing spondylitis in South Korea: A national health insurance database study

**DOI:** 10.1371/journal.pone.0240155

**Published:** 2020-10-06

**Authors:** Jin-Sung Park, Jae-Young Hong, Hak-Kyu Kim, Bongmo Koo, Sang-Hee Kim, Yong-Chol Kwon

**Affiliations:** 1 Department of Orthopedics, Samsung Medical Center, Sungkyunkwan University School of Medicine, Seoul, South Korea; 2 Department of Orthopedics, Korea University Ansan Hospital, Ansan-si, Gyeonggi-do, South Korea; 3 Medical Affairs, Pfizer Pharmaceuticals Korea Ltd., Seoul, South Korea; Karolinska Institutet, SWEDEN

## Abstract

No studies of the current status of treatment options are available for ankylosing spondylitis **(**AS) patients in South Korea. This study assesses the current status of AS treatment trends using a nationwide database. This study was conducted using a Korean National Health Insurance System (KNHIS) dataset from 2006 to 2016. We randomly extracted 50% of the total number of patients registered as As patients in the KNHIS. The distribution of the number of patients according to age and gender was analyzed each year. The types and combination methods of drugs used during the study period were estimated yearly. Between 2006 and 2016, the number of AS patients increased linearly by an average of 9% annually, 6372 in 2006 to 15188 in 2016. The study found that the use of nonsteroidal anti-inflammatory drugs (NSAIDs) was the most commonly prescribed pharmacological treatment option, followed by disease-modifying anti-rheumatic drugs (DMARDs) and then biologics. Biologics such as tumor necrosis factor alpha (TNF-α) inhibitors increased from 10% to 35% consistently for 10 years. In terms of combination therapy, DMARDs + NSAIDs accounted for almost 90% of treatments in 2006, but decreased by 65% in 2016. The use of biologics and NSAIDs increased from 3% to 28%. Prescriptions for dual therapies and mono therapies largely dominated prescription habits, accounted for up to approximately 80% of treatments. Among 10- to 14-year-old patients, there was no triple therapy, dual and triple therapies decreased gradually for those 60 and older, and the ratio of conservative treatments has increased. This study shows how South Korea reflects changes in AS treatment trends, along with the emergence of TNF-α inhibitors that are effective in treating AS. Research on clinical outcomes for AS treatments will be needed on following these drug changes.

## Introduction

Ankylosing spondylitis (AS), the most common form of spondyloarthopathies, is a chronic inflammatory disease that causes spinal stiffness, deformity, and postural defects [1]. Other manifestations include peripheral arthritis, enthesis, dactylitis, and pain in the hip and buttock region [2]. Inflammatory enthesopathy progressing to ossification and ankyloses is the pathologic basis for the disease [3].

AS usually presents in the third decade of life and rarely after the age of 45. It typically follows a chronic progressive course [4], with a life-long impact on patients [5]. Prevalence varies according to differences in genetics, ethnicity, and environmental factors [6]. The overall prevalence of AS is between 0.1% and 1.4% [7]. The primary goal of AS treatment is symptomatic and inflammatory control, and the prevention of progressive structural damage to help maintain and normalize quality of life. The current recommended treatments include daily nonsteroidal anti-inflammatory drugs (NSAIDs), including Coxibs (COX-2 inhibitors) as first-line therapies for patients with AS suffering from pain and stiffness [8]. Systemic glucocorticoids are not recommended [9], although local glucocorticoid injections are considered for localized musculoskeletal inflammation [10]. More recently, tumor necrosis factor alpha (TNF-α) inhibitors, such as adalimumab, infliximab, or etanercept, have been found to be effective for patients with peripheral and axial symptoms [11, 12].

Accurate estimates of treatment trends are important when planning health care policies, but no studies of the current status of treatment options have been conducted in AS patients in South Korea. The purpose of this study is to confirm the number of AS patients and assess the current state of drug therapy between 2006 and 2016 using a nationwide database.

## Materials and methods

### Data sources

This study was based on a Korean National Health Insurance System (KNHIS) dataset from 2006 to 2016. The KNHIS covers 97% of the population and allowing patients to pay just 30% of total healthcare costs. The remaining 3% of the population is the lowest-income households, and the Medical Aid Program covers all their medical expenses. Healthcare institutions submit claims for the remaining 70% of the total medical cost to the government. Medical information on almost all patients in healthcare institutions is prospectively integrated into the KNHIS claim database, which includes extensive information on diagnoses and comorbidity codes classified by the 10th revision of the International Classification of Diseases (ICD-10), demographic characteristics, admission and ambulatory care, prescription records, and procedure codes.

### Data collection and analysis

To analyze the trend of AS patient's drug use, 50% of the total number of patients registered as AS patients in the KNHIS was randomly extracted and analyzed. In the KNHIS, patients were diagnosed with AS if they met the modified New York criteria [13]. These clinical criteria include: 1) low back pain and stiffness for at least 3 months that was improved by exercise and not relieved by rest; 2) limitation motion of the lumbar spine in both the sagittal and frontal planes; and 3) limitation of chest expansion relative to values normal for age and sex. The radiological criterion is grade ≥ II bilateral sacroiliitis or grade III to IV unilateral sacroiliitis. Definite AS is diagnosed if the radiological criterion plus 2 of the 3 clinical criteria are present. The ICD-10 codes for AS (M45.0 to M45.9) were used in the KNHIS data.

Therefore, all analyzes used 50% of all AS patients in South Korea provided by KNHIS. In order to show demographic characteristics of the data, the distribution of the number of patients according to age and gender was analyzed each year. We assessed the prescription patterns of NSAIDs, disease-modifying anti-rheumatic drugs (DMARDs) and biologics, which are the three main therapeutic classes of drugs (by series) for AS, in accordance with Assessment of SpondyloArthritis International Society/European League Against Rheumatism (ASAS/EULAR) treatment guidelines [9]. For the analysis of drug use, the main pharmaceutical substance code was used. The main substances for each drug are as follows. Celecoxib, Meloxicam, Aceclofenac, Naproxen, Ibuprofen, Dexibuprofen, Ketoprofen, Diclofenac, Etodolac, and Piroxicam were used for NSAID; sulfasalazine and methotrezate were used for DMARDs; Etanercept, infliximab, Adalimumab, Golimumab, and Secukinumab were used for Biologics. The types and combination of drugs used during the study period were estimated. Data analyses were conducted with SAS version 9.3 (SAS Institute, Cary, NC, USA).

### Ethics statement

This study was approved by the Institutional Review Board of Korea University, Ansan Hospital. (IRB No. AS17143). The study protocol was approved by the KNHIS Institutional Review Board. An informed-consent exemption was granted by the board.

## Results

### Demographic characteristics of the study group

Between 2006 and 2016, the number of AS patients increased linearly by an average of 9% annually, 6372 in 2006 to 15188 in 2016. The number of AS patients temporarily decreased from 2009 to 2010 because AS was included as the Rare Intractable Disease (RID) in 2009 and KNHIS reaffirmed the strict diagnosis for AS. The age of onset in both men and women was highest in those aged 30 to 39 years. The number of men with AS was 3.8 times higher than the number of women, and significantly higher across all age groups (Table 1).

**Table 1:** Distribution of AS patients according to age and gender from 2006 to 2016.

Year	2006	2007	2008	2009	2010	2011	2012	2013	2014	2015	2016
**Number**	6372	7080	7926	8589	7967	9036	10059	11128	12485	13763	15188
**Mean age**	38.2 ± 13.8	38.3 ± 13.4	39.1 ± 13.6	39.7 ± 13.7	39.0 ± 12.4	39.6 ± 12.5	40.2 ± 12.6	40.8 ± 12.8	41.4 ± 13.1	42.3 ± 13.7	43.0 ± 13.9
**0–9 (%)**	3 (0.05)	2 (0.03)	5 (0.06)	0 (0)	0 (0)	0 (0)	0 (0)	1 (0.01)	2 (0.02)	3 (0.02)	0 (0)
**10–19 (%)**	190 (2.98)	199 (2.81)	207 (2.61)	200 (2.33)	173 (2.17)	218 (2.41)	211 (2.1)	214 (1.92)	217 (1.74)	247 (1.79)	259 (1.71)
**20–29 (%)**	1739 (27.29)	1808 (25.54)	1893 (23.88)	1845 (21.48)	1649 (20.70)	1717 (19.0)	1799 (17.88)	1893 (17.01)	2107 (16.88)	2269 (16.49)	2422 (15.95)
**30–39 (%)**	2046 (32.11)	2381 (33.63)	2625 (33.12)	2935 (34.17)	2799 (35.13)	3070 (33.98)	3322 (33.03)	3557 (31.96)	3793 (30.38)	3789 (27.53)	3979 (26.2)
**40–49 (%)**	1196 (18.77)	1357 (19.17)	1601 (20.2)	1772 (20.63)	1810 (22.72)	2134 (23.62)	2455 (24.41)	2811 (25.26)	3191 (25.56)	3610 (26.23)	3999 (26.33)
**50–59 (%)**	589 (9.24)	695 (9.82)	819 (10.33)	967 (11.26)	962 (12.07)	1223 (13.53)	1440 (14.32)	1660 (14.92)	1965 (15.74)	2262 (16.44)	2571 (16.93)
**60–69 (%)**	378 (5.93)	429 (6.06)	491 (6.19)	525 (6.11)	411 (5.16)	481 (5.32)	592 (5.89)	706 (6.34)	839 (6.72)	1063 (7.72)	1337 (8.8)
**70–79 (%)**	201 (3.15)	175 (2.47)	241 (3.04)	284 (3.31)	136 (1.71)	164 (1.81)	205 (2.04)	238 (2.14)	302 (2.42)	411 (2.99)	485 (3.19)
**80– (%)**	30 (0.47)	34 (0.48)	44 (0.56)	61 (0.71)	27 (0.34)	29 (0.32)	35 (0.35)	48 (0.43)	69 (0.55)	109 (0.79)	136 (0.9)
**Sex (Male%)**	4991 (78.33)	5604 (79.15)	6271 (79.12)	6766 (78.78)	6433 (80.75)	7261 (80.36)	8060 (80.13)	8890 (79.89)	9906 (79.34)	10751 (78.12)	11807 (77.74)

### Prescription patterns

**Fig 1:**
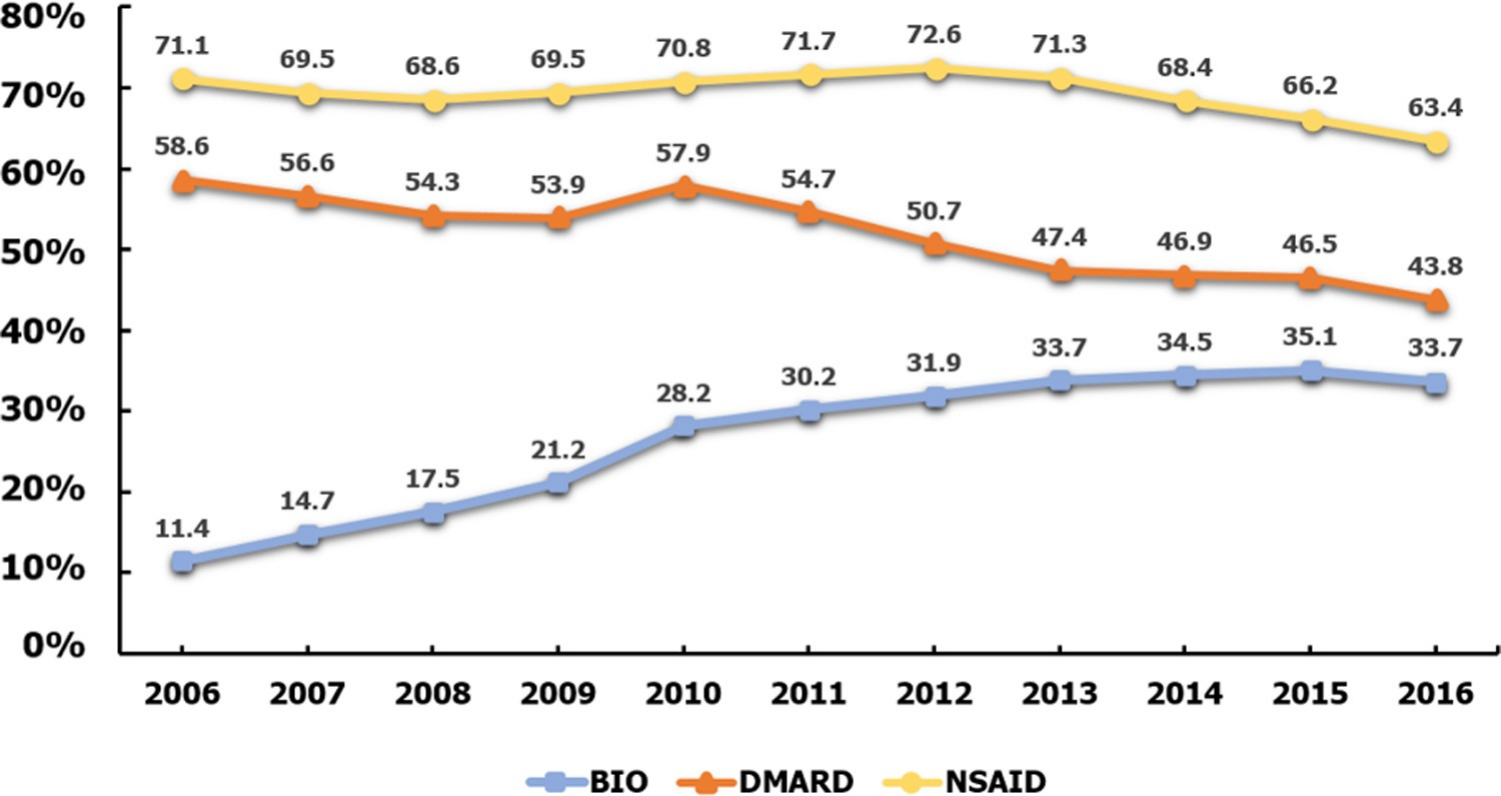
Prescription rate of NSAIDs, DMARDs, and biologics by year.

NSAIDs were the most prescribed modes of pharmacotherapy of the commonly prescribed drug classes, and prescription habits remained largely consistent throughout the 10-year period. Prescriptions for DMARDs, though lower than NSAIDs, peaked in 2010 and then dropped significantly to nearly half the number of prescriptions by 2016. Conversely, prescriptions for biologics increased steadily from 2006 to 2016 (Fig 1).

**Fig 2:**
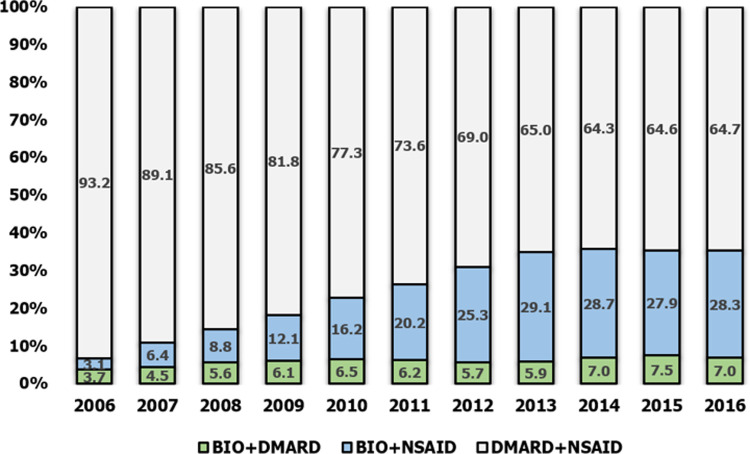
Pattern of drug prescriptions for dual therapies by year.

The prescription of combination therapies was largely dominated by NSAIDs + DMARDs options, which accounted for an average of three-quarters of all combination prescriptions. However, prescriptions for such combinations decreased steadily from 2006 to 2013, after which it stabilized at approximately 64% of all treatments. Prescriptions for a combination of a biologic + NSAIDs increased steadily from 2006 to 2013, and then remained constant from 2013 to 2016 at an average of 20.3% of all combination prescriptions over the 10-year period. The combination of a biologic + DMARDs remained below 10% of all prescriptions over the decade, with a slight increase in the last three years (Fig 2).

**Fig 3:**
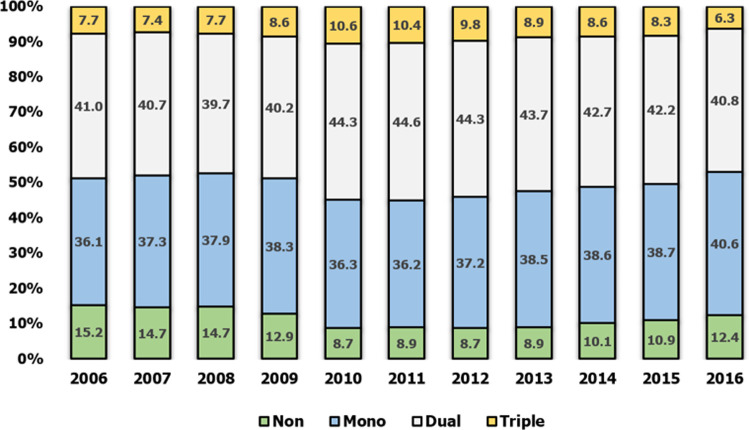
Prescription patterns for combination therapies by year.

**Fig 4:**
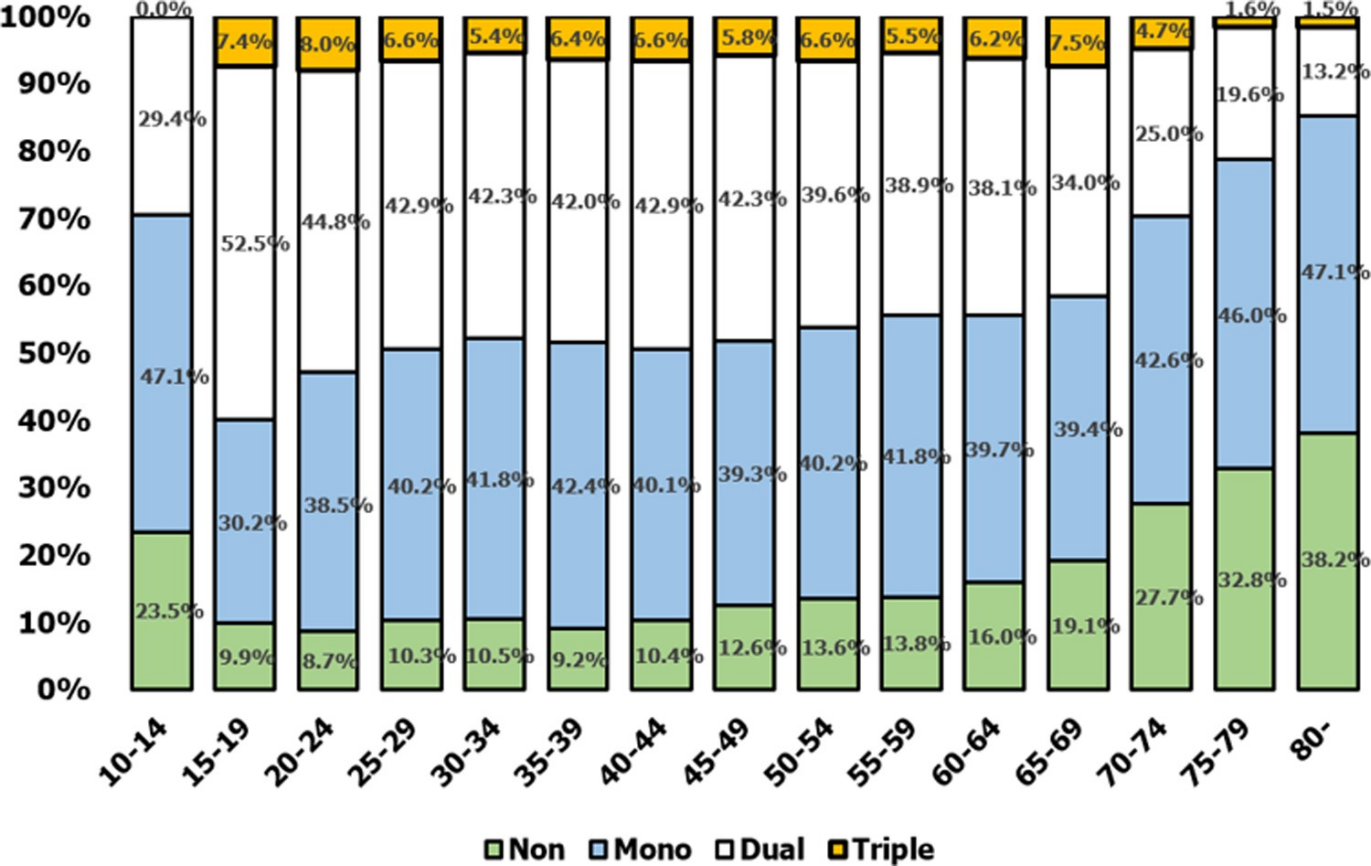
Prescription patterns of combination therapies by age.

Prescriptions for dual therapies and mono therapies largely dominated prescription habits. These accounted for approximately 80% of the share of prescriptions across the decade (Fig 3). Prescription patterns by age (Fig 4), though largely dominated by prescriptions for mono and dual agents, showed more variation. In the 10–14 age group, monotherapy was prescribed in nearly half the cases (47.1%) and dual therapy in 29.4% of cases, with the remaining cases involving a conservative treatment other than these pharmacotherapy agents. In the 15–19 age group, more than half the prescriptions were for a dual therapy (52.5%) as a triple therapy began to appear. From 20 to 60 years of age, dual- and triple-therapy prescription rates were similar. However, after the age of 60, dual and triple therapies decreased gradually and the ratio of conservative treatments increased.

## Discussion

The aim of this study was to identify patterns in drug therapy for AS in South Korea. The results clearly demonstrate that NSAIDs were the most commonly prescribed pharmacological treatment options, followed by DMARDs and then biologics. Biologics increased from 10% to 35% consistently for 10 years. In terms of combination therapy, DMARDs + NSAIDs accounted for almost 90% of treatments in 2006, but decreased by 65% in 2016. Meanwhile, the use of biologics and NSAIDs increased from 3% to 28%.

In the past, the choice of drugs in AS patients was limited to NSAIDs and DMARDs [14, 15]. Recently, biologics such as TNF-α inhibitors have been reported to be effective for skeletal AS manifestations such as spinal and sacroiliac pain, peripheral arthritis, and enthesitis [11, 16]. However, while DMARDs play an important role in the treatment of rheumatoid arthritis (RA), the effects on axial inflammation in AS patients have not been clearly demonstrated [17, 18]. The most commonly used sulfasalazine among DMARDs is associated with some benefits in reducing peripheral joint symptoms, but no benefit has been found with respect to physical function, pain and spinal mobility [17, 18]. The 2010 ASAS/ELAR guidelines recommend the use of biologics for axial symptoms, regardless of DMARDs use if there is no proper response to NSAIDs. For peripheral joint symptoms, biologics are recommended if one local corticosteroid injection does not produce a response or if a DMARD such as sulfasalazine does not have the intended effect [9].

Treatment trends for AS patients in other countries also show that the use of biologics has increased dramatically. In Germany, the use of TNF-α inhibitors increased by 53% from 2000 when the use of TNF-α inhibitors was approved in 2012, and the combination of an NSAID + TNF-α inhibitors, accounted for 32% of treatments, which is the largest proportion [19]. Although the use of TNF-α inhibitors is increasing in Korea, this study shows that DMARDs use remains high. The first reason for this is insurance standards. In South Korea, drugs are rarely used without insurance because all populations are covered by health insurance. Currently, insurance for biologics covers only cases for which two or more NSAIDs or DMARDs have been used for more than three months but the treatment was not effective or was stopped due to side effects. The number of patients with AS in Korea shows that the symptoms of peripheral arthritis may influence the high rates of DMARDs use [20]. In addition, 70% to 80% of patients with AS can control their symptoms with an NSAID alone [8]. Finally, some small and local hospitals may find it difficult to manage the adverse effects associated with the new biologics.

TNF inhibitors are key mediators against infection [21]. Previous meta-analyses of randomized controlled trials reported that infection risks increased after the use of TNF-α inhibitors in RA patients [22]. The most important adverse effect of using TNF-α inhibitors in AS patients is infection, and if the immune system is weak, TNF-α inhibitors can be a burden. For similar reasons, it is easy to understand why triple therapy accounts for roughly 10% of prescriptions: drug toxicity or other adverse reactions must be balanced against treatment efficacy. In this study, considerable variation was observed with regards to prescription patterns by age. Up to 15 years and beyond 75 years of age, the number of triple-therapy prescriptions is negligible. This may indicate intolerance of triple therapy at vulnerable ages. Similarly, the highest prevalence of no pharmacotherapy can be observed up to 15 years of age and then between the ages of 75 and 85. These data are suggestive of weakened immune systems that cannot tolerate any form of medication.

However, many recent papers report that the use of TNF-α inhibitors in AS patients does not significantly increase serious adverse events, including infection, and is safer previously assumed [23]. Studies have already shown that the use of TNF-α inhibitors can be more effective than DMARDs such as sulfasalazine, and recent guidelines recommend the use of TNF-α inhibitors over sulfasalazine in patients with peripheral arthritis, unless otherwise contraindicated [24]. Overall, the use of TNF-α inhibitors appears to be unnecessarily low in South Korea.

This study was limited by a lack of information on which medications were used depending on the degree of symptoms and changes in clinical symptoms after the use of the medication. It incorporates information about the ratio and pattern of medications, but not the continuity of medications. Therefore, no information on which drugs are used continuously and on how the usage patterns of the drugs have changed sequentially was included. Finally, information for drugs used as uninsured methods was not included.

## Conclusion

This study shows how drug use patterns in AS patients have changed over the past decade. In particular, it shows how South Korea reflects changes in AS treatment trends along with the emergence of TNF-α inhibitors as effective treatment options. Further research on clinical outcomes of AS treatments will be needed following these developments.
